# Highly Substituted 10-RO-(hetero)acenes—Electric Properties of Vacuum-Deposited Molecular Films

**DOI:** 10.3390/molecules28176422

**Published:** 2023-09-03

**Authors:** Bernard Marciniak, Sylwester Kania, Piotr Bałczewski, Ewa Różycka-Sokołowska, Joanna Wilk, Marek Koprowski, Jacek Stańdo, Janusz Kuliński

**Affiliations:** 1 Structural & Material Chemistry Group, Faculty of Science and Technology, Institute of Chemistry, Jan Dlugosz University in Częstochowa, Armii Krajowej 13/15, 42-200 Częstochowa, Poland; b.marciniak@ujd.edu.pl (B.M.); piotr.balczewski@cbmm.lodz.pl (P.B.); e.sokolowska@ujd.edu.pl (E.R.-S.); 2 Centre of Mathematics and Physics, Łódź University of Technology, Żeromskiego 116, 90-924 Łódź, Poland; sylwester.kania@p.lodz.pl; 3 Faculty of Technical Physics, Information Technology and Applied Mathematics, Żeromskiego 116, 90-924 Łódź, Poland; jacek.stando@p.lodz.pl; 4 Functional Materials Synthesis Group, Division of Organic Chemistry, Centre of Molecular and Macromolecular Studies, Polish Academy of Sciences, Sienkiewicza 112, 90-363 Łódź, Poland; skalik1984@wp.pl (J.W.); marek.koprowski@cbmm.lodz.pl (M.K.)

**Keywords:** hole mobility, electric properties, anthracene, benzo[b]carbazole, aromatic carbaldehyde, DFT calculations, quantum-chemical calculations, vacuum-deposited molecular film

## Abstract

The functionalization of the aromatic backbone allows the improvement of the electrical properties of acene molecules in the amorphous layered structures of organic thin films. In the present work, we discuss the electric properties of the stable, amorphous, vacuum-deposited films prepared from five highly substituted 10-RO-acenes of various electronic properties, i.e., two extreme electron-donor (1,3-dioxa-cyclopenta[b]) anthracenes with all RO substituents, two anthracene carbaldehydes and one benzo[b]carbazole carbaldehyde possessing both electron-donor and acceptor substituents. The hole mobility data were obtained using subsequent steady state space charge limited currents (SCLC) and Time of Flight (TOF) measurements, performed on the same sample and these were then compared with the results of theoretical hole mobility calculations obtained using the Density Functional Theory (DFT) quantum—chemical calculations using the Marcus–Hush theory. The study shows a good agreement between the theoretical and experimental values which allows for the quick and quantitative estimation of Einstein’s mobility values for highly substituted 10-RO anthracene and benzo[b]carbazole based on chemical calculations. This agreement also proves that the transport of holes follows the hopping mechanism. The theoretical calculations indicate that the reorganization energy plays a decisive role in the transport of holes in the amorphous layers of highly substituted hetero(acenes).

## 1. Introduction

**Figure 1:**
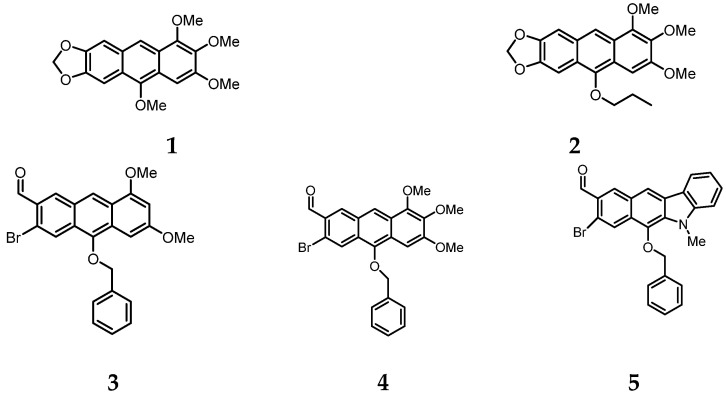
Chemical structures of two electron-rich anthracenes with all donor, cyclic and acyclic alkoxy substituents, i.e., 1,2,3,10-tetramethoxy-6,7-(1,3-dioxa-cyclopenta[b])anthracene (**1**) and 10-*n*-butoxy-1,2,3-trimethoxy-6,8-(1,3-dioxa-cyclopenta[b])anthracene (**2**) as well as three electron donor-acceptor acenes, i.e., 10-(benzyloxy)-3-bromo-6,8-dimethoxyanthracene-2-carbaldehyde (**3**), 10-benzyloxy-bromo-6,7,8-trimethoxyanthracene-2-carbaldehyde (**4**) and 10-benzyloxy-8-bromo-5-methyl-5*H*-benzo[b]-carbazole-9-carbaldehyde (**5**).

Linearly fused aromatic and heteroaromatic hydrocarbons, called (hetero)acenes, are an important group of compounds for applications in molecular optoelectronics. Unsubstituted or low-substituted acenes are the best known, but there is a lack of knowledge and general synthetic methods regarding higher-substituted analogues [1,2,3,4,5,6]. In addition, the more fused rings acenes contain (as pentacenes and higher analogs), the lower their stability. Therefore, acenes with a lower number of fused rings, such as anthracenes, but with a higher number of electron-diverse substituents, appear to be an ideal solution that can provide optimal molecular stability and satisfactory electrical and photoluminescent properties. For the synthesis of highly substituted (hetero)acenes containing up to 4–6 diverse substituents, we have developed the *hetero*-Friedel-Crafts-Bradsher reaction in three variants [1,2,3,4,5,6]. We employed the *oxo*-variant to synthesize 10-RO-(hetero)acenes **1**–**5** for the present studies (Figure 1).

Although intensive research has been carried out on the properties of unsubstituted and low substituted acenes, such as anthracene derivatives, for applications in organic field effect transistors (OFETs) [7,8,9,10], there is a lack of contributions devoted to charge carrier transport and the measurement of electrical properties in highly substituted acene systems, such as **1**–**5** [6].

In this context, there is a need to elaborate fast procedures for obtaining quantitative approximations concerning the charge carriers mobilities of the amorphous intrinsic materials. This work compares the charge carriers mobility data obtained from experimental measurements with theoretical predictions, based on density functional theory (DFT) calculations at the B3LYP level of theory, for 10-RO-acene molecules in the gas phase [11,12,13,14]. The quantities that determine charge transport, such as HOMO, LUMO levels, reorganization energy, transfer integral and hopping rate, may be determined using calculations with use of the quantum chemistry package Gaussian 09 [14,15].

In this study, experimental data were obtained using a series of subsequent steady state space charge limited currents (SCLC) and the Time of Flight measurements made on the same samples. The proper value of the hole drift mobility was determined with the use of TOF measurements. By varying the I-U characteristics obtained from SCLC measurements using spline interpolation (DM-SCLC), quantitative values for the microscopic parameters of the hole transport were obtained. 

The empirical determination of the plausible charge transport mechanism is usually realized in the layered structures. The theoretical explanation of the mechanism of electric charge transport for molecular materials must take into account strong covalent bonds within the molecule and much weaker intermolecular interactions. This mechanism, described under the Marcus–Hush theory, is known as hopping [12,13,14,15]. In this study, we show that hopping is the leading hole transport mechanism for highly substituted 10-RO-acenes. We compare experimentally obtained charge transport data, such as drift mobility, Einstein mobility electric conductivity and concentrations of trapping states, with the Einstein mobility calculated using DFT calculations in the scope of the Marcus–Hush theory.

## 2. Results

### 2.1. X-ray Diffraction (XRD)

**Figure 2:**
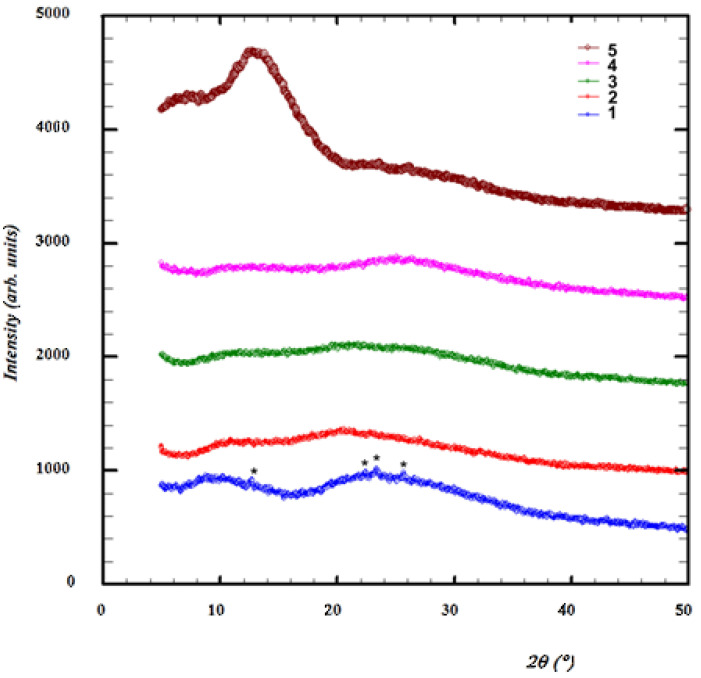
X-ray diffraction patterns registered for obtained layers of acenes **1**–**5**. Asterisks indicate reflections from the crystalline phase of the acene **1**.

An analysis of the XRD diffractograms recorded for layers of **1**–**5** (Figure 2) generally showed that they were all amorphous. The exception to this was layer **1** where, in addition to the amorphous halo, four weak reflections were noticeable at 2θ = 12.68, 22.42, 23.36 and 25.68°. They corresponded to the positions of the reflections (2θ = 12.53, 21.34, 23.43 and 26.44°) on the theoretical diffractogram calculated from the crystal structure of **1** (CSD refcode: CAPBAY [1]). Thus, the features revealed on the diffraction pattern of the layer of **1** could be attributed to the coexistence of two phases in the layer of **1**, i.e., an amorphous phase and a crystalline phase.

The thickness of the tested layers for diffraction tests was 1 μm–5 μm for anthracenes **1**–**4**, and 3 μm for the benzo[b]carbazole **5**.

The method of thermal vacuum deposition used in this work requires the high thermal stability of molecules suitable for making the “sandwich” cell. The anthracene and benzo[b]carbazole derivatives **1**–**5** selected for the study exhibited a thermal stability sufficient to obtain measurement cells. All measurements discussed in this work were carried out at temperatures near 300 K, under ambient atmosphere and at 50–55% humidity.

The roughness of the layers was determined using the AFM technique (Atomic Force Microscope). AFM images (height mode) were obtained using an AFM Nanoscope IIIa MultiMode 5 (molecular resolution) with table heated to 250 °C (Veeco, Plainview, NY, USA). Surface roughness was determined using the Nanoscope software.

Typical AFM images of the evaporated layers, made under the conditions described in this article, are presented in reference [1] (**3**–**5**). For layers **3**–**5**, the surface roughness (R_ms_) was below 50 nm, and for layers **1** and **2** it was below 80 nm, when the measured line in the AFM image was 5 μm long.

### 2.2. Analysis of the Results of the Electric Measurements

#### 2.2.1. Spline Interpolation of TOF Measurements

**Figure 3:**
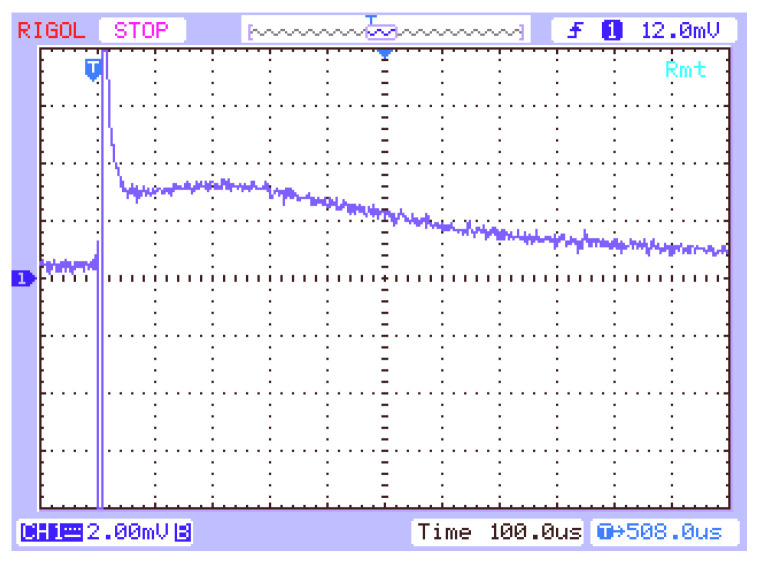
The oscillogram I-t for **3** (U = 20 V, L = 8.25 μm).

**Figure 4:**
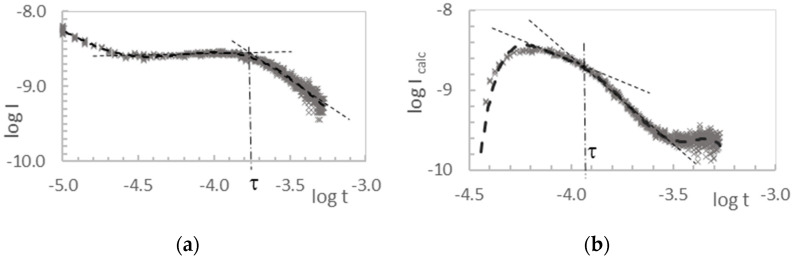
The logI-logt characteristics for layer **3** (U = 20 V, L = 8.25 μm): without taking into account the effect of the RC constant of the circuit (**a**); when taking into account the effect of the RC constant of the circuit (**b**).

The purpose of measuring the current–time characteristics I-t was to determine the time-of-flight (τ_TOF_) as a function of the polarization voltage (U), or the lifetime of the charge carriers (τ_f_). The effect of the RC time constant of the measurement system on the shape of the obtained pulse was eliminated, as was described in reference [16]. An example of a current–time pulse, obtained directly on the oscilloscope screen and then presented in a double-logarithmic form, and after taking into account the effect of the RC time constant of the measurement system for the hole conduction of layer **3**, is shown in Figure 3 and Figure 4. The value of the RC time constant of the measurement system was 170 μs. The waveform mentioned above, after elaboration using the spline method and after transformation to the logU—logt system, but without taking into account the effect of the RC time constant, is shown in Figure 4a. The hole transit time determined by the graphical method in this case is τ_f_ = 10^−3.76^ s = 174 μs. The same oscillogram after processing with consideration of the influence of the RC time constant of the measurement circuit is shown in Figure 4b. In this case, the determined hole flight time is smaller and is τ_calc_ = 10^−3.93^ s = 117 μs.

#### 2.2.2. DM-SCLC Analysis of SCLC Measurements

**Figure 5:**
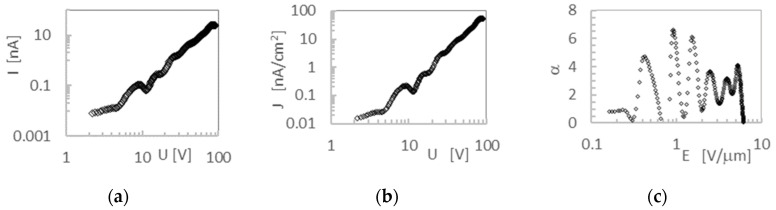
Transformation of the steady state characteristics for layer **4**: the I-U characteristics in log I–log U system (**a**); the J-U characteristics presented in logJ–logU system (**b**); the α-E characteristics (**c**).

**Figure 6:**
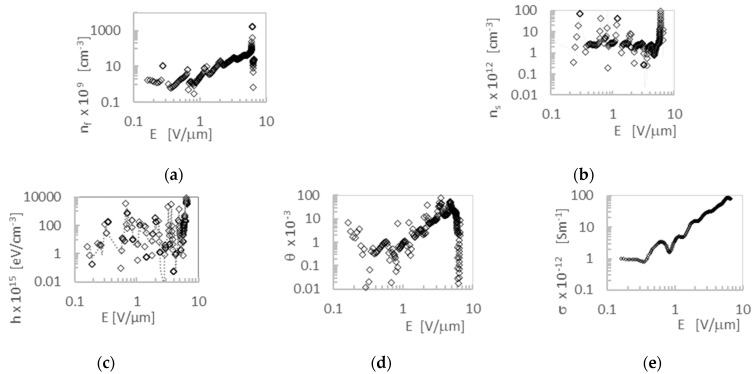
Dependences of electric properties of layer **4** on electric field strength: for free carrier concentration n_f_ (**a**); for the trap concentration n_s_ (**b**); for density of states h (**c**); for the trap filling coefficient θ (**d**); for the electric conductivity σ (**e**).

The graph of the characteristics of steady state dark currents I-U and J-U has been transformed to the α-E coordinate system in the manner shown in Figure 5. DM-SCLC analysis, described by formulas (2)–(5), allowed the obtaining of the dependences of the concentration of free carriers (n_f_), the concentration of traps (n_s_), the density of trap states (h), the trap filling coefficient (θ), and the specific conductivity (σ), as a function of electric field strength (E). The result of the calculations carried out for **4** is shown in Figure 6. For all other compounds, a similar nature of the relationship was obtained.

A summary of the results of the steady state dark currents measurements (SCLC measurements), using the spline interpolation and the differential method (DM-SCLC) on the tested compounds, is shown in Table 1. 

### 2.3. Analysis of the DFT Calculations

The electrical properties of the layers that depend on the geometry of the molecule are primarily associated with the spatial electron density distribution of the molecule in the neutral, ionized and excited states. The exchange of electrons between adjacent molecules is determined by the spatial distribution of the frontier molecular orbitals and their energy levels for the neutral molecules, i.e., HOMO and LUMO orbitals and the value of band gap E_g_. Quantum-chemical calculations allow for the calculating of the properties of any chosen quantum system, including those that cannot be directly studied using experimental methods. The calculation results allowed an analysis of the impact of substituents on the properties of molecules. Table 2 presents the energy values of the frontier molecular orbitals (FMO) for the studied anthracenes **1**–**4** and the benzocarbazole **5**. Table 3 contains, for comparison, the calculated energy values for non-functionalized naphthalene, anthracene, tetracene and carbazole. The calculated energy values for HOMO and LUMO levels and the value of the energy gap, E_g_, for non-substituted compounds, in conjunction with the known properties of substituents, would allow the drawing of a conclusion regarding the possibility of intentionally modifying the electrical properties of materials through the proper selection of donor or acceptor functional groups.

## 3. Discussion

The amorphous packing of the tested layers prevents the formation of privileged conduction directions and positional disorders and is the source of many trap levels (Figure 5c). This is seen as the variability of density function (h) with respect to the field (E) applied to the sample (Figure 6c). The statistical distribution of state density near the HOMO level is the reason that the average value of the concentration of free carriers (n_f_), and the average value of traps (n_s_) involved in transport, does not show strong variability. This is despite the presence of statistical fluctuations in the positions of the molecules. 

The anthracenes **1**–**4** and the benzo[b]carbazole **5** showed similar reorganization energies calculated for the hole transport (Table 4). The amorphous structure of the layers and their robustness against ambient conditions could indicate the possible use of such materials as active layers in photovoltaic cells (OPVs) [17,18]. The conduction process at a microscopic scale can be considered as a combination of red-ox processes occurring sequentially as charge carriers transfers between neighboring molecules. A problem that may arise in electronic and optoelectronic applications is the chemical instability of the layers during conduction, related to the deformation of the molecules and their packing caused by the transfer of charge carriers. However, the repeatability of the I-U and I-t characteristics obtained during experiments carried out on the layers of anthracenes **1**–**4** and the benzo[b]carbazole **5** leads to the conclusion that the structure of the layers did not change qualitatively during conduction. 

Moreover, data from the literature indicate that the compounds studied in this work meet the sufficient conditions for robustness against oxygen and moisture during electrical measurements, due to the suitable values of HOMO and LUMO levels [19,20,21]. Materials conducting positive charge carriers with a HOMO energy level below −5 eV are considered as sufficiently stable for manufacturing organic electronics devices [19]. All the anthracenes **1**–**4** and the [b]carbazole **5** studied in this work meet this condition. For anthracene and benzo[b]carbazole derivatives **1**–**5**, HOMO levels range from −5.742 eV for **5** to −5.119 eV for **4**. For the hole conductivity, the most favorable energy value of the HOMO level is above −5.5 eV. Above the level of −5 V, the material may be susceptible to oxidation under the presence of atmospheric oxygen during hole conduction [22]. The rate of the spontaneous degradation of semiconductor materials under atmospheric conditions (oxygen, water vapor) is related to the values of the LUMO level. If the value is below −4 eV then there is no danger associated with the oxidation process [23]. If the value of the LUMO level is higher, but close to the −4 eV value, the degradation process is slow. For example, a p-type OFET transistor using an active pentacene layer, whose HOMO level is −4.56 eV and LUMO level is −2.39 eV, shows in air, without the use of protective layers, the stability of the electrical parameters over a period of at least three months [24]. On the other hand, the OFET transistor built on the dinaphtho [2,3-b:2′,3′ f]thieno [3,2-b]thiophene (DNTT) layer, whose HOMO level is −5.4 eV and LUMO is −2.4 eV, shows a negligible loss of electrical parameters and negligible hysteresis of these parameters over a long period of time [25,26].

**Figure 7:**
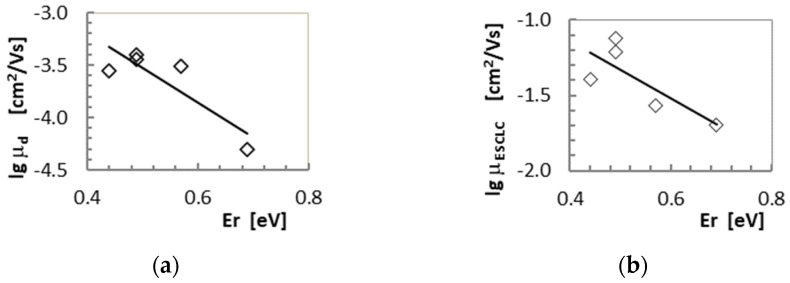
Dependence of (**a**) the drift mobility from TOF measurements and (**b**) the Einstein mobility calculated from SCLC measurements as a function of reorganization energy for measured materials.

Among the materials studied in this work, anthracene and carbazole derivatives, such as **4** (−2.582 eV), **3** (−2.770 eV) and **5** (−2.348 eV), have convenient LUMO levels. The aforementioned compounds also have the lowest forbidden gap energy values in the range from 3.035 eV to 3.394 eV, clearly lower than for the other two compounds studied. Shinamura et al. [26] report that an E_g_ value of 3.0 eV is considered very convenient for the operation of electronic devices based on organic materials. The LUMO values of all the compounds studied in this work are above −4 eV, which may indicate that the materials studied do not meet the conditions necessary for the appearance of electron conduction in an organic material [23]. However, the values of the HOMO level indicate that hole conduction dominates in the studied materials. DFT calculations show a clear dependence on the experimentally determined drift mobility and the Einstein mobility on the reorganization energy for all the studied compounds (Figure 7, Table 4 and Table 5).

**Figure 8:**
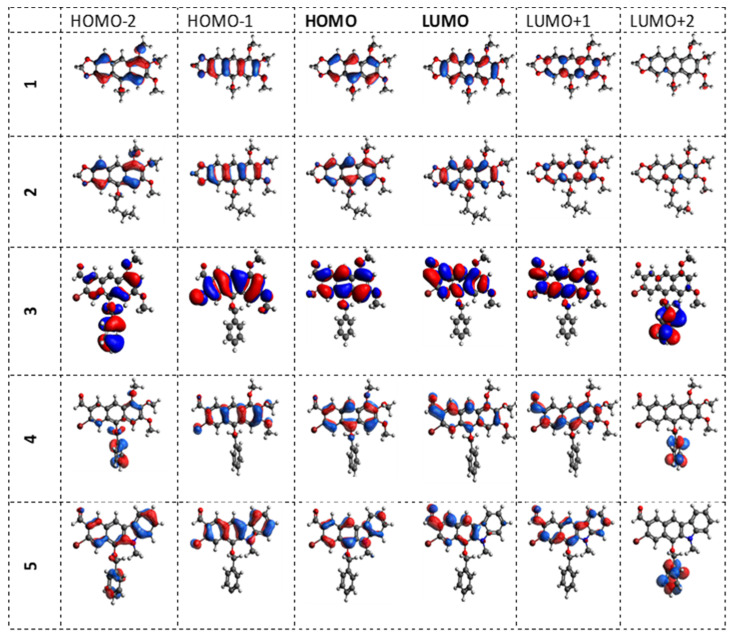
Geometry of the distribution of HOMO and LUMO orbitals for the studied compounds including HOMO, HOMO-1,HOMO-2, LUMO, LUMO+1 and LUMO+2 levels.

On the other hand, no clear correlation is observed between the calculated transfer integral and the calculated charge transfer rates and the mobility value (Table 4). This indicates the existence of additional mechanisms affecting the transport process than just the reorganization energy and matching energy levels. The lack of such a relationship is most likely due to the fact that, in the amorphous layers, the probability of carrier transfer between neighboring molecules depends on the disordered distance of the molecules and the disordered nature of their intermolecular interactions [27] as well as the different spatial distribution of FMO orbitals that is, HOMO and LUMO. This is clearly visible in Figure 8. The shape of the HOMO orbitals is similar for all the derivatives studied. However, the shape of the HOMO-1 orbital is clearly different for each of the derivatives studied. Therefore, it seems that the geometric factor in the packing of molecules is very important here in the process of transferring holes between molecules. Similar relationships are also observed by other authors [28].

## 4. Materials and Methods

The measurements of electric properties were made using the time-of-flight (TOF) and space charge limited currents (SCLC) methods. SCLC measurements need to obtain current–voltage characteristics using the constant–current method [29,30,31,32]. Obtaining unambiguous results of the electrical parameter values that can be obtained from the analysis of the SCLC measurement data requires knowledge of the drift mobility. The drift mobility values sought here are provided by TOF measurements [12,16,33].

The measurements for each sample consisted of a sequential measurement of the I-U characteristics followed by the TOF measurement. Data analysis, with use of cubic spline approximations, made it possible to differentiate the obtained I-U characteristics (DM-SCLC method) which allowed the determination of the microscopic parameters defining the electric transport of holes. The measurements were made in the layered “sandwich” structure (Figure 4). The polarization voltage needed to perform the measurements was applied to outer metallic electrodes. 

### 4.1. Synthesis of Organic Materials 

The (hetero)acenes **1**–**5** were synthesized using the *oxo*-variant of the Friedel-Crafts-Bradsher reaction, developed in our lab [1,2].

### 4.2. Preparation of Samples for Testing by Vacuum Evaporation Method 

The thermal vacuum evaporation method was used to produce thin film layers. This method ensures a good purity of the obtained layers and the absence of solvent molecules. Due to the weak van der Waals interactions between the molecules of the low-molecular-weight materials under study, the evaporation temperatures of organic materials are much lower than those required in the technological processes for obtaining inorganic semiconductors. In practice, evaporation temperatures are below 600 K. Such a low temperature of the evaporation process allows a reduction in the influence of additional external factors on the quality of the obtained layer. 

The thermal vacuum evaporation method ensures a good electrical contact between the measured layer and the measuring electrodes. The bottom electrode, made of Au, was deposited onto a glass substrate beforehand. The process of making it does not affect the performance of the proper hydrocarbon layer. However, a higher deposition temperature is required for the deposition of the upper Al electrode to ensure adequate aluminum vapor pressure in the deposition chamber. In the case of the tested compounds, the metal electrodes attached to the hydrocarbon measured layers formed a mechanically stable system and did not delaminate during evaporation or later during measurements of electrical properties. Quartz round plates (d = 32 mm/1 mm thick) were used as substrates. The thermal evaporation of the sensing electrodes was carried out under a vacuum of 10^−5^ Tr. The vacuum evaporation of Au- and Al-sensing electrodes was carried out from a tungsten wire, upon which the evaporated Au electrode material of 4 N purity and Al of 5 N purity was placed. The thermal evaporation of the organic layer was conducted from a quartz crucible. The substrate temperature was about 300 K, and the deposition rate was about 10 nm/s. The choice of substrate temperature corresponded to the temperature of conducting electrical measurements.

### 4.3. XRD Measurements

The X-ray diffraction (XRD) method was used to evaluate the structural properties of the examined layers. The X-ray patterns of thin layers of the investigated hydrocarbons were recorded using a HZG-4 powder diffractometer Seifert GmbH Ost (Hannover, Germany) with independent controller Dronek (Krakow, Poland) (CuKα radiation) working in the Bragg–Brentano geometry. The recording was carried out in the angle range of 2θ = 5–50° with a scan step of 0.02°. The thickness of the tested layers for diffraction tests was 1 μm–5 μm for anthracenes **1**–**4**, and 3 μm for the benzo[b]carbazole **5**. The thickness of the layers was determined with an accuracy of ±0.5 μm using a Digital Micrometer IP5 just after vacuum deposition.

### 4.4. Measurements of Electric Properties

**Figure 9:**
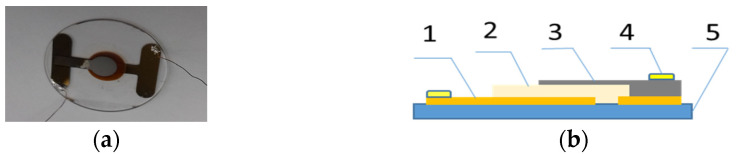
A view of the measurement cell in the layered structure (**a**) and a cross-section of the measurement cell (**b**) (1—Au electrode, 2—examined layer, 3—Al electrode, 4—In contact, 5—glass substrate).

The investigations of electrical properties of the organic materials **1**–**5** were carried out using a measuring cell, in which the metal-organic material-metal layers formed “sandwich” structures. A layer of organic material (3) was deposited onto a glass substrate (5) equipped with a Au electrode (1). A semitransparent Al layer (3) was directly deposited onto the organic layer and metal contacts (4), made of In, were soldered in. The resulting measuring cell formed a planar capacitor with a plate area of typically about 0.5 cm^2^ (Figure 9). During the electrical measurements, the measuring cell was placed in a Faraday cage, which allowed the avoiding of the influence of electromagnetic interference on the measurement result.

For cells prepared in this way, their electrical capacitance C was measured using a Semi-Automatic RLC Bridge type E314 and the thickness of organic layer was evaluated using the following formula:

(1) $$L=\frac{\varepsilon \varepsilon_{0}S}{C}$$

where L—thickness of the organic layer, ε—relative dielectric constant of the layer, ε_0_—vacuum dielectric constant, S—surface area of the electrode and C—the measured capacitance. According to references [25,26], a value of relative dielectric constant ε = 3 was taken for calculations. 

Hole mobility measurements, obtained using subsequent steady state space charge limited currents (SCLC) measurements and Time-of-Flight (TOF) measurements, were performed on the same sample. The measurements of the described compounds obtained in the form of amorphous layers were conducted at room temperature and a humidity of 45–55%. 

#### 4.4.1. SCLC Measurements

**Figure 10:**
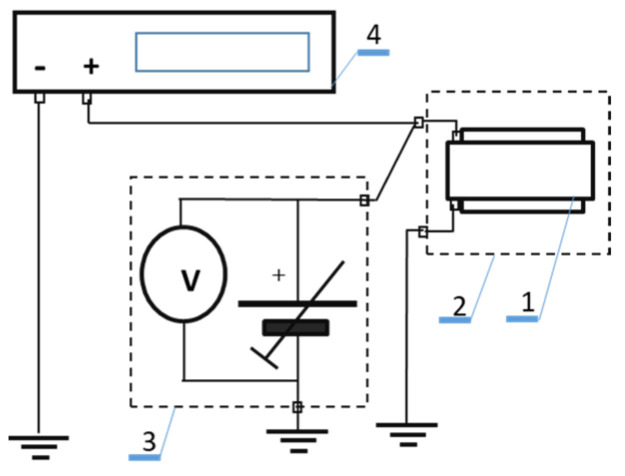
Measurement system for determining dark DC (I-U) characteristics: 1measurement cell, 2-Faraday cage, 3-low noise power supply, 4-electrometer.

The I-U constant–current characteristics were determined using the measurement system shown in Figure 10. The measuring cell was created as a system of flat “sandwich” layers (Figure 9). The determination of the I-U constant–current characteristic makes it possible to obtain the dependence of the current variation as a function of the layer polarization voltage over a very wide range of values. The current passing through the layer during the measurement usually varied by several decades, i.e., from values of the order of 10^−12^ A to values of 10^−7^ A. During the measurement, the sample was sequentially passed through a range of ohmic currents, space-charge-limited currents with traps and space-charge-limited currents without traps. The measurement results revealed the different internal mechanisms occurring during conduction inside the organic semiconductor sample.

#### 4.4.2. TOF Measurements

The time-of-flight (TOF) test method is a commonly used method for studying the transport properties of charge carriers for materials characterized by low conductivity and low charge carriers mobility, such as disordered organic materials and polymers with π-type coupling [10,12,16,33]. A drift mobility measurement, in order to determine the properties of charge carriers of one type, requires the generation of excess charge in the layer, which will be transferred to the electrodes by interacting with the electric field inside the layer. This is achieved either by the sudden injection of charge carriers into the material layer, or by inducing a rapid charge separation inside the layer. If one sets the measurement conditions so that the process of formation of unbalanced charge carriers in the layer is faster than the time of transfer of these carriers to the electrode, the possibility of measuring the time of flight of carriers through the layer or the lifetime of these carriers appears. The thickness of the organic layer L should be as large as possible, also the influence of the RC constant of the circuit should be taken into account. In turn, this can minimize the measurement signal to unmeasurable values. The procedure for carrying out the TOF measurement requires, successively: applying an electric field, generating charge carriers, transporting charge carriers through the organic layer and recording the image of instantaneous currents on the screen of a digital oscilloscope.

**Figure 11:**
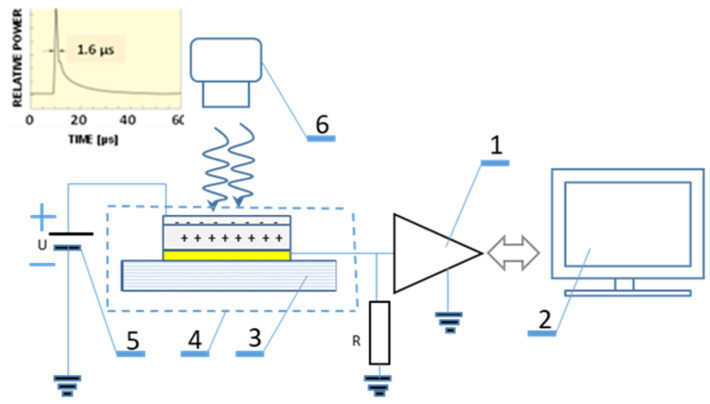
Measurement system for testing instantaneous photocurrent I-t. 1—digital oscilloscope, 2—computer, 3—measuring cell, 4—Faraday cage, 5—low-noise power supply, 6—pulsed UV lamp.

The measurement of instantaneous currents through the layer, defined by the functional relation I = f(t,U), was carried out using the measurement system (Figure 11). The measuring cell (3) was pre-polarized with a DC voltage from a low-noise power supply (5), so that the organic layer was polarized in the reverse direction and the field inside the layer was homogeneous. A flash of UV light (6), passing through the semi-transparent electrode and short in comparison to the duration of the measured transient current, caused charge separation near the semi-transparent electrode. The transporting electric field E, created by polarizing the measuring cell with a voltage U_B_ supplied by a low-noise power supply (5), caused charge carriers to drift inside the examined layer of organic material as the thin sheet transported through the layer. The carriers were extracted at the collecting electrode. The instantaneous current passing through the measuring cell forced a potential drop across the resistor R_L_ connected in series with the sample. The instantaneous current was recorded using a digital oscilloscope (1) and the entire waveform was recorded with a computer (2), which controlled the signal acquisition.

### 4.5. DM-SCLC Calculations

The theoretical calculations of electrical properties, such as mobility of charge carriers μ, concentration of charge carriers n_f_, concentration of traps n_s_, trap filling coefficient θ and density of trap states N_t_, with the use of the DM-SCLC method, were carried out based on the results obtained by measuring current–voltage characteristics using the constant–current method [30,31,32] and current–time characteristics using the instantaneous current measurement method [12,16,33].

To determine the parameters of charge carrier transport, the theoretical calculation procedure of the differential method (DM SCLC) was used. The calculations needs determine the third derivative of the obtained waveform I-U. A significant facilitation in the analysis of the results is the use of the approximation of the obtained I-U characteristics by interpolation using the cubic spline method. 

The calculation procedure of the differential method (DM SCLC) allows the calculation of the values of the concentration of free carriers n_f_, the concentration of traps n_s_, the density of trap states h and the level of trap filling coefficient θ. Decisive in obtaining good quality results is the determination of three consecutive continuous derivatives of the U-I waveform, i.e., the determination of coefficients α, α′, α″. These coefficients were calculated after transforming the I-U characteristics to J-U characteristics (where J is obtained after converting the experimentally obtained value of I using the relation J = I/A, where J—current density and A—electrode area) presented on a double logarithmic scale, lnJ-lnU:

(2) $$\alpha =\frac{d\left(lnJ\right)}{d\left(lnU\right)},\;\alpha \prime =\frac{d^{2}\left(lnJ\right)}{d{\left(lnU\right)}^{2}},\;\alpha \prime \prime =\frac{d^{3}\left(lnJ\right)}{d{\left(lnU\right)}^{3}}$$

The continuity required for the calculations for the obtained waveform is provided using the method of cubic splines for calculation of α, α′, α″. Cubic splines minimize the bending of the line passing through two adjacent two-dimensional points of the experimentally determined current–voltage characteristics, and they provide continuity of the first and second derivatives of each point at which the arcs of polynomials approximating the experimental points are combined.

The DM-SCLC analysis of such transformed experimental data uses a set of the following equations:

(3) $$n_{f}=\frac{\alpha }{2\alpha -1}\frac{JL}{e\mu U}$$

(4) $$n_{s}=\frac{\rho_{L}}{e}=\frac{2\alpha -1}{\alpha }\frac{\alpha -1}{\alpha }\left[1-\frac{\alpha \prime }{\alpha \left(2\alpha -1\right)\left(\alpha -1\right)}\right]\frac{\varepsilon \varepsilon_{0}U}{eL^{2}}$$

(5) $$\theta =\frac{\alpha^{4}}{{\left(2\alpha -1\right)}^{2}\left[\alpha \left(2\alpha -1\right)\left(\alpha -1\right)-\alpha \prime \right]}\frac{JL^{2}}{\mu \varepsilon \varepsilon_{0}U^{2}}$$

where e is the charge of the electron, μ is the drift mobility of the charge carrier (this work uses the value of drift mobility determined experimentally using the TOF method), L is the thickness of the sample, ε_0_ is the dielectric constant of the vacuum, ε is the relative dielectric constant of the material, n_f_ is the concentration of free charges on the cathode, n_s_ is the concentration of carriers trapped on states located near the cathode and L is the space charge limiting the current flow. Gaining knowledge of the values of the parameters, as determined in this way, makes it possible to determine the values of charge carrier mobility and conductivity. 

The study of dark currents, that is, current–voltage characteristics, was carried out using the measurement system, as shown in Figure 10. The dependence of the current flowing through the layer, I, as a function of the applied voltage, U, as a result of these measurements was obtained. The I-U characteristics obtained in this way were transformed to the logJ-logU characteristics (J current density). After applying the spline method, it was transformed to the characteristics α-E (E electric field density inside the layer, α slope of the characteristics determined by formula (2), α = d(logJ)/d(logU)). The α-E characteristics obtained in this way were used to determine the values of the concentration of free carriers n_f_, the concentration of traps n_s_, the density of trap states h and the trap filling coefficient θ, using the differential DM SCLC method.

### 4.6. DFT Calculations

Theoretical calculations for isolated molecules were made using density functional theory (DFT) performed with use of Gaussian 09. The frontier molecular orbitals (FMO) properties, i.e., HOMO and LUMO orbitals and the values of organization energy (Er), were determined, as in the works of Datta et al. [34] and Deng et al. [35], where the Marcus [36] and Hush [37] theories were used to determine the nature of the charge transport process [38,39,40]. The results of the abovementioned calculations made it possible to analyze the character of the charge transfer process in a manner similar to that presented by Deng et al. [35], Ren et al. [41], Mendels et al. [42], Campbell [43] and Rossi [44]. 

## 5. Conclusions

The electric properties of vacuum-deposited, stable amorphous films, prepared from highly substituted 10-RO-acenes, were measured for the first time. 

The reorganization energy appears to be the determining factor for the carrier mobility of the derivatives under study (Figure 7).

An analysis of the currents at the equilibrium state shows that, for fields in the range of 10^6^ Vm^−1^ to 10^7^ Vm^−1^, the electrical properties of the studied materials are stable and do not show strong variation, which allows the use of the layers of these materials in optoelectronic devices. The structure of the molecules in which there are oxygen-containing substituents may favor conductivity due to the presence of relatively weakly bound electron pairs on the oxygen atoms. These electrons can actively influence the charge distribution in aromatic rings.

The relatively high value of the Einstein hole mobility offers the possibility of using these materials under conditions of strong carrier generation. The low drift mobility of the studied materials and, at the same time, the weak dependence on conductivity of the layers on the applied electric field, also favors the application of these materials in optoelectronics.

The studied anthracene and carbazole derivatives have a suitable arrangement of FMO energy levels for efficient hole transport. The energy values of the HOMO and LUMO levels lead to the conclusion that the studied compounds have a good environmental stability

The two methods presented in this paper for obtaining Einstein mobility values, i.e., the first based on experimental measurements, in which the determination of the degree of filling of the traps is an important element, and the second based only on DFT theoretical calculations, gave similar values for these quantities. Such agreement was obtained within the framework of the Marcus–Hush theory, which assumed hopping transport as the dominant mode of transport. The above results, in a technological sense, are useful.

The presented study is included in the scope of works on the important role of the appropriate functionalization of molecules in the process of searching for the optimal layer properties used for the needs of organic electronics. The roughness Rmn of the layers obtained in our article is of a similar relative value, in relation to the thickness of the obtained layers, to those obtained in technological studies [45,46,47], i.e., below 5%.

It is interesting for us that high vacuum techniques are essential in obtaining materials for organic electronics. The results, presented in references [45,46,47], obtained in polycrystalline layers indicate the dependence of the obtained parameters on the grain size. This is an indication for us that amorphous layers should favor the electrical stability of the layers.

It is clear that the computational procedure developed allows us to quickly estimate the Einstein mobility values for highly substituted anthracenes and carbazoles. We previously obtained similar results for naphthalene derivatives [12]. Such a use of DFT calculations can be helpful in the search for materials that meet the requirements of organic electronics and optoelectronics. The presented results confirm the validity of the choice of the Marcus–Hussian theory for modeling the transport of electric current carriers. The procedure presented in the paper provides an opportunity to quickly evaluate promising molecular structures.

## Figures and Tables

**Table 1:** Electrical properties of the studied compounds **1**–**5**. The calculations were based on measured data performed in the range of SCLC currents, electric conductivity (σ), maximum concentration of free charge carriers that can be obtained by thermal emission (n_0_), density of trapping states (h) and concentration of the localized states (N_v_).

Compound	σ	n_0_	h	N_v_
	[S m^−1^]	[cm^−3^]	[eV^−1^ cm^−3^]	[cm^−3^]
**1**	2.0 × 10^−12^–5 × 10^−9^	(9.4 ± 1.5) × 10^14^	(4.8 ± 1.4) × 10^15^	(1.1 ± 0.4) × 10^15^
**2**	6.0 × 10^−14^–7.2 × 10^−11^	(3.6 ± 1.1) × 10^14^	(6.0 ± 1.8) × 10^15^	(1.2 ± 0.4) × 10^15^
**3**	1.3 × 10^−14^–7.7 × 10^−11^	(9.6 ± 2.9) × 10^12^	(5.2 ± 1.6) × 10^18^	(8.0 ± 2.4) × 10^14^
**4**	7.1 × 10^−14^–1.2 × 10^−13^	(4.7 ± 1.4) × 10^13^	(2.5 ± 0.8) × 10^16^	(5.9 ± 1.8) × 10^15^
**5**	2.0 × 10^−12^–5.0 × 10^−9^	(3.1 ± 0.9) × 10^13^	(1.0 ± 0.3) × 10^14^	(4.5 ± 1.4) × 10^14^

**Table 2:** Energy levels of FMO for studied compounds at the B3LYP/6-311++G(d,p) level.

Compound	Frontier Orbital Energy Level		Energy Gap
	E_HOMO_, [eV]	E_LUMO_, [eV]	E_g_ = E_LUMO_ − E_HOMO_, [eV]
**1**	−5.150	−1.592	3.558
**2**	−5.119	−1.569	3.550
**3**	−5.638	−2.533	3.105
**4**	−5.682	−2.582	3.100
**5**	−5.742	−2.348	3.394

**Table 3:** Energy levels of FMO for non-functionalized naphthalene, anthracene, tetracene and carbazole at the B3LYP/6-311++G(d,p) level.

Compound	Frontier Orbital Energy Level		Energy Gap
	E_HOMO_, [eV]	E_LUMO_, [eV]	E_g_ = E_LUMO_ − E_HOMO_, [eV]
naphthalene	−6.147	−1.398	4.749
anthracene	−5.576	−2.024	3.552
tetracene	−5.142	−2.401	2.741
carbazole	−5.822	−1.155	4.667

**Table 4:** The values of reorganization energy (E_r_), charge transfer integral (J_ij_) and charge transfer rate (K_e_) for studied compounds at the B3LYP/6-311++G(d,p) level.

Compound	E_r_	J_ij_	Ke
	[eV]	[eV]	[Hz]
**1**	(0.49 ± 0.13)	(0.35 ± 0.17)	(2.5 ± 2.1) × 10^13^
**2**	(0.49 ± 0.13)	(0.36 ± 0.18)	(2.7 ± 2.2) × 10^13^
**3**	(0.57 ± 0.14)	(0.53 ± 0.27)	(2.5 ± 2.0) × 10^13^
**4**	(0.69 ± 0.17)	(0.48 ± 0.24)	(6.2 ± 4.2) × 10^12^
**5**	(0.44 ± 0.11)	(0.35 ± 0.17)	(4.2 ± 3.3) × 10^13^

**Table 5:** Hole drift mobility (μ_d_), measured by TOF, trap filling coefficient (θ) calculated from SCLC measurements and the Einstein mobility derived from measurements (μ_Ed_) and calculated theoretically from DFT calculations (μ_E_).

	TOF Measurements	from SCLC Data	from TOF and SCLC Data	Calculated Theoretically from DFT
Derivative	μ_d_	θ	μ_Ed_	μ_E_
	[cm^2^/(Vs)]		[cm^2^/(Vs)]	[cm^2^/(Vs)]
**1**	(3.6 ± 1.4) × 10^−4^	(6.0 ± 1.8) × 10^−3^	(6.1 ± 2.7) × 10^−2^	(1.1 ± 0.4) × 10^−2^
**2**	(4.0 ± 1.6) × 10^−4^	(5.3 ± 1.6) × 10^−3^	(7.5 ± 3.3) × 10^−2^	(2.3 ± 0.9) × 10^−2^
**3**	(3.1 ± 1.2) × 10^−4^	(1.1 ± 0.3) × 10^−2^	(2.7 ± 1.2) × 10^−2^	(2.2 ± 0.9) × 10^−2^
**4**	(5.0 ± 3.2) × 10^−5^	(2.5 ± 0.8) × 10^−3^	(2.0 ± 0.9) × 10^−2^	(5.3 ± 2.1) × 10^−2^
**5**	(2.8 ± 1.8) × 10^−4^	(7.0 ± 2.1) × 10^−3^	(4.0 ± 1.8) × 10^−2^	(3.6 ± 1.4) × 10^−2^

## Data Availability

Not applicable.

## Sample Availability

Samples of the compounds are not available from the authors.
